# Bactericidal behavior of silver nanoparticle decorated nano-sized magnetic hydroxyapatite

**DOI:** 10.1039/d4na00183d

**Published:** 2024-09-14

**Authors:** Ebrahim Sadeghi, Reza Taghavi, Amir Hasanzadeh, Sadegh Rostamnia

**Affiliations:** a Cellular and Molecular Research Center, Cellular and Molecular Medicine Research Institute, Urmia University of Medical Sciences Urmia 57157-89400 Iran hasanzadeh.a@umsu.ac.ir; b Organic and Nano Group, Department of Chemistry, Iran University of Science and Technology Tehran 16846-13114 Iran rostamnia@iust.ac.ir srostamnia@gmail.com

## Abstract

Methicillin-resistant *Staphylococcus aureus* (MRSA) is the most common cause of acute bacterial arthritis. Due to the increase in antibiotic resistance in these bacteria, the discovery of new antibacterial agents has become one of the hot topics in the scientific community. Here, we prepared a nano-sized porous biocompatible magnetic hydroxyapatite through a solvothermal method. Then, we adopted a post-synthesis modification strategy to modify its surface for the stabilization of Ag NPs through a green reduction by the euphorbia plant extract. Moreover, the results show that the prepared composite perfectly prevents the aggregation of Ag NPs. This composite was used as a bactericidal and antibiofilm agent against MRSA bacteria in an *in vitro* environment, which showed excellent results. Also, the cell viability assay indicates that the prepared composite has low cytotoxicity, making it a perfect antibacterial agent for *in vivo* experiments.

## Abstract 2

Methicillin-resistant *Staphylococcus aureus* (MRSA) is the most common cause of acute bacterial arthritis.

## Graphical abstract

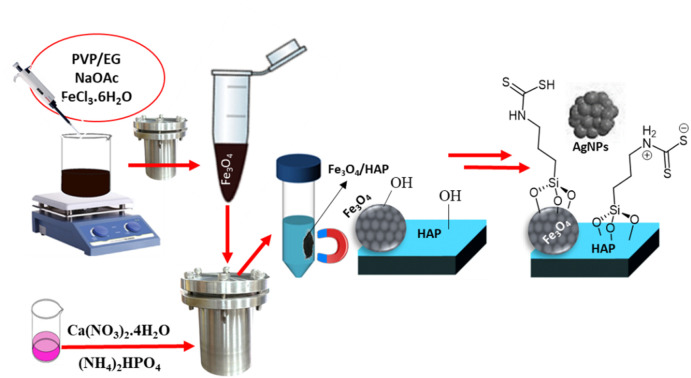

## Introduction

Acute bacterial arthritis is an emergency infectious disease. Bacterial proliferation in the joint and subsequent inflammatory process can lead to rapid local joint destruction and may be associated with systemic infection. Methicillin-resistant *Staphylococcus aureus* (MRSA) is the most common cause of infectious arthritis.^1^ Therefore, the implementation of appropriate targeted therapy is necessary to limit the morbidity and mortality associated with these infections. Considering the high resistance of bacteria causing acute arthritis to available antibiotics, alternative drugs must be used to eliminate these resistant bacteria.^2^

The emergence of antibiotic-resistant bacteria underscores the importance of finding alternative bactericidal agents.^3^ In this respect, a high surface area-to-volume ratio and the intrinsic antibacterial properties of Ag NPs make them a perfect candidate against Gram-positive and Gram-negative bacterial strains.^4^ Studies show that Ag NPs can deactivate multidrug-resistant (MDR) bacteria in combination with conventional antibiotics or alone by destroying the bacteria's defense system.^5,6^ It is well established that the bactericidal properties of silver NPs highly depend on their size, shape, charge, and dosage. The main drawback in the practical application of Ag NPs is their agglomeration in solution.^7–9^ To prevent the aggregation process, the implantation of solid heterogeneous materials as supports for the stabilization and heterogenization of the NPs is proposed as a valid method.^10^ Such supports not only lead to the heterogenization and formation of monodisperse NPs but can modify the bactericidal properties of the NPs.^11^ Biological barriers including biofilms are also affected by AgNPs. A biofilm is an aggregation of microorganisms that live within a matrix comprised of an extracellular polymeric substance (EPS). It seems that the biofilm increases the resistance of microbes to antibiotics as well as poor nutrients, desiccation, ultraviolet radiation, *etc.* The Minimum Inhibition Concentration (MIC) of microbes inside the biofilm is higher and they need higher therapeutic concentrations. AgNPs disrupt the biofilm structure by binding to the exopolysaccharide matrix, which finally leads to cell lysis and death.^12–14^

Hydroxyapatite (HAP), one of the most appreciated bioceramics, has recently gained tremendous attention in biomedical applications owing to its similarity to bone structure.^15,16^ Such resemblance leads to its superior biocompatibility and bioactivity compared to conventional ceramics. Moreover, the porous nature of HAP paves the way for its application as a support for the stabilization of nanoparticles.^17^ The resulting composite will possess biocompatible pores with enhanced bactericidal properties.

Magnetization of materials is considered a judicious method to increase their reusability and decrease the operation cost by reducing material waste.^18,19^ Moreover, the biocompatibility of iron oxides along with their ability to be controlled by an external magnetic field led to the broad interest of medical scientists in them.^20^ Furthermore, the ease of surface modification facilitates their combination with various materials which leads to their broad application in various fields, including biomedicine, catalysis, environmental, photocatalysis, photothermal treatments, *etc.*^21^ The small size of magnetic NPs also helps their retention in blood circulation.

Here, we prepared a porous biocompatible magnetic hydroxyapatite material as a viable support for the stabilization of Ag NPs. The surface of the magnetic composite is functionalized with the dithiocarbamate functional group to produce a suitable surface for the stabilization of Ag NPs. Reduction of Ag NPs by the euphorbia plant extract was selected as a green and eco-friendly method for the reduction of Ag NPs. The results showed that the designed composite perfectly prevents the aggregation of Ag NPs, which leads to its superior performance as an antibacterial agent. Moreover, the cell assay experiments indicate that the Fe_3_O_4_/HAP-NHCS_2_H.Ag_NPs_ show low cytotoxicity, making them a perfect candidate for *in vivo* experiments.

## Experimental

### Materials and reagents

All the materials and reagents were obtained from Sigma Aldrich and were used without any further purification.

### Synthesis of magnetic Fe_3_O_4_

The synthesis of Fe_3_O_4_ was performed by adding 1.5 g FeCl_3_·6H_2_O, 2 g NaOAc, and 1 g PVP to 25 mL of ethylene glycol. The subsequent solution was stirred for 2 h until complete dissolution. The resulting yellow mixture was transferred to a Teflon-lined stainless-steel autoclave (50 mL) and heated at 200 °C for 10 h. After cooling to ambient temperature, the sediment was collected using a magnet, washed several times with distilled water and ethanol, and dried for 12 hours in a vacuum at 60 °C.

### Preparation of magnetic hydroxyapatite

The preparation of magnetic hydroxyapatite nanoparticles was carried out by synthesizing HAP in the presence of Fe_3_O_4_ NPs. In this context, 5 mL aqueous solution containing 0.5 g Fe_3_O_4_ and 3.37 mmol Ca(NO_3_)_2_·4H_2_O and another 5 mL aqueous solution containing 2 mmol (NH_4_)_2_HPO_4_ were added to a round bottom flask. The pH of the solution was adjusted to 11 and the solution was vigorously stirred for 30 min. The resulting solution was refluxed at 90 °C for 2 h and then incubated at room temperature for 24 h. The sediment was separated using an external magnet, washed with deionized water several times, and dried at 90 °C overnight. Finally, the crude product was ground with a mortar and pestle and stored for subsequent use.

### Silanization of magnetic hydroxyapatite

First, 0.5 g Fe_3_O_4_@HAP was dispersed in a 30 mL solution of APTES in toluene (37 mmol L^−1^) by ultrasonication for 10 min. The resulting solution was stirred at 40 °C for 48 h. The sediment was separated from the solution using an external magnet, washed with toluene several times, and dried in an oven at 60 °C to prepare Fe_3_O_4_/HAP/PrNH_2_.

### Preparation of Fe_3_O_4_/HAP/PrNHCS_2_H

The obtained sediment from the previous step was suspended in 20 mL methanol under sonication for 10 min. Next, 2.2 mL CS_2_ was added to this solution and stirred for 12 h at room temperature. The resulting product was separated from the mixture using an external magnet, washed with ethanol, and dried at 70 °C overnight.

### Collection and preparation of the euphorbia plant extract

Aerial organs of the euphorbia plant were collected during the flowering stage and dried under the shadow. Green branches of the plant were ground after drying and were used for further use. 100 g of the ground powder was added to a beaker and enough pure EtOH was added to the beaker to soak the powder. After 24 h, the powder was separated from the mixture by filtration, and the excess EtOH was evaporated to collect the pure extract.

### Stabilization of Ag NPs

First, 0.12 g Fe_3_O_4_/HAP/PrNHCS_2_H was dispersed in 30 mL deionized water ultrasonically for 15 minutes. Then, the solution was transferred to a water bath and mechanically stirred for 5 minutes at ambient temperature. 100 mL AgNO_3_ solution with appropriate concentration was added and stirred for another 5 minutes. Subsequently, the Euphorbia plant extract solution was added to the above solution dropwise for 10 minutes under vigorous stirring and stirred for 5 hours at room temperature. Finally, the supernatant was discarded and the precipitate was collected and thoroughly washed with deionized water. The final product was dried in an oven at 60 °C. The concentration of the Ag solution was regulated to prepare composites with 3, 7, and 10 wt% of Ag NPs. Throughout this manuscript, the term Fe_3_O_4_/HAP/PrNHCS_2_H.Ag_NPs_ refers to the composite with 7% of Ag NP content.

### Sampling and antibacterial activity assay

10 samples of *Staphylococcus aureus* isolated from patients with infectious arthritis were collected from the microbiology laboratory of Shahid Motahari Hospital in Urmia, and confirmed by additional tests.^22^ All isolates were methicillin-resistant *Staphylococcus aureus*. Next, the antibacterial properties of the Fe_3_O_4_/HAP/PrNHCS_2_H.Ag_NPs_, including MIC (minimum inhibitory concentration) and MBC (minimum bactericidal concentration), were evaluated by the broth dilution method. Also, for comparison, the vancomycin antibiotic was used to evaluate the sensitivity of clinical isolates, and the methicillin-resistant *Staphylococcus aureus* (ATCC 43300) was used as the control strain. Finally, the inhibition of bacterial biofilm formation in the presence of the target compound was evaluated by the microtiter plate method.^23^ All the above methods were performed according to the Clinical and Laboratory Standards Institute (CLSI) guidelines.

### Cell viability assay

NIH-3T3 (mouse embryonic fibroblast cell line) in RPMI medium with fetal bovine serum (FBS) (10% (v/v)), and 0.5 mL of penicillin–streptomycin solution with a final concentration of 50 IU mL^−1^ penicillin and 50 (μg mL^−1^) streptomycin (Sigma-Aldrich), under standard conditions (5% CO_2_, 37 C and 95% humidity), was cultured. After that, the cells were treated with different concentrations of Fe_3_O_4_/HAP/PrNHCS_2_H.Ag_NPs_ (0, 25, 50, 100, 200 ppm) for 24 h incubation. Then, the MTT test (3-(4,5 dimethylthiazol)-2-diphenyltetrazolium bromide) was used to evaluate cell viability.^11^ Finally, using a microplate reader, cell viability was measured at 570 nm (absorbance value).

## Results and discussion

**Scheme 1:**
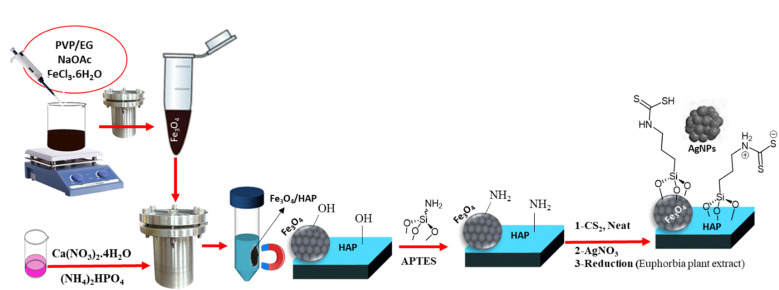
Schematic illustration of the preparation steps involved in the preparation of magnetic HAP.

Nano-sized magnetic core–shell Fe_3_O_4_/HAP/PrNHCS_2_H was carefully prepared by adopting a step-by-step post-synthesis modification strategy. First, magnetic Fe_3_O_4_ NPs have been prepared through a solvothermal method and used as a core for the growth of the HAp shell. To prepare a suitable support to stabilize the Ag NPs, dithiocarbamate functionalities are decorated over the surface of the magnetic composite. For this reason, silanization of the magnetic HAp was performed in the presence of APTES, and the amine functionalities of the resulting composite were transformed into dithiocarbamate functional groups by adding CS_2_ to the composite. The sulfur functionalities of the composite prepared a superior support for the stabilization of the Ag NPs and not only prevented the aggregation of the NPs but increased their efficiencies (Scheme 1).

**Fig. 1:**
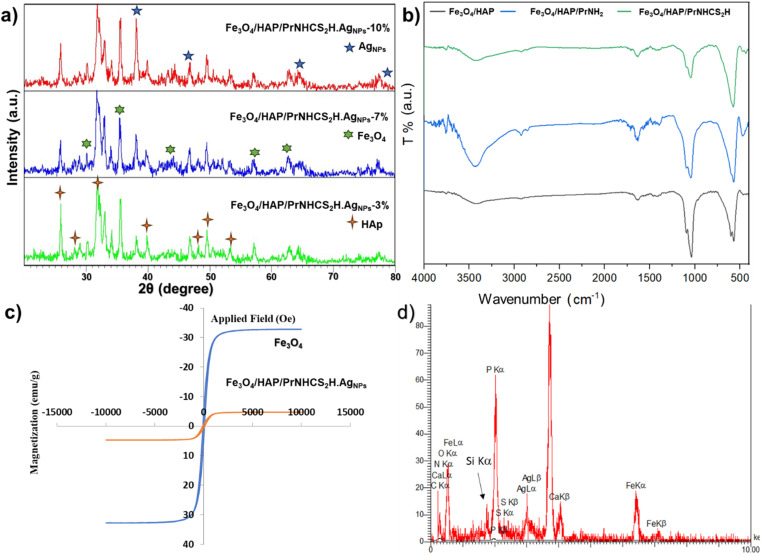
(a) The XRD patterns of the Fe_3_O_4_/HAP/PrNHCS_2_H.Ag_NPs_ with 3, 7, and 10% of Ag loading. (b) The FT-IR spectra of the Fe_3_O_4_/HAP, Fe_3_O_4_@HAp-NH_2_, and Fe_3_O_4_/HAP/PrNHCS_2_H. (c) The VSM plots of the bare Fe_3_O_4_ and Fe_3_O_4_/HAP/PrNHCS_2_H.Ag_NPs_. (d) EDS image of the Fe_3_O_4_/HAP/PrNHCS_2_H.Ag_NPs_.

Fig. 1a shows the XRD patterns of the Fe_3_O_4_/HAP/PrNHCS_2_H.Ag_NPs_ with 3, 7, and 10% of Ag loading. The expected patterns of Fe_3_O_4_ and HAp are obvious in all the presented XRD patterns. All the provided XRD patterns show the 220, 311, 400, 511, and 440 miller indices of Fe_3_O_4_, indicating the formation of Fe_3_O_4_ NPs. As specified in Fig. 1a, the XRD patterns of the Fe_3_O_4_/HAP/PrNHCS_2_H.Ag_NPs_ show the 002, 210, 211, 310, 222, 213, and 004 miller indices of HAp. Moreover, the characteristic peaks of the Ag NPs are evident in all three XRD patterns, with an increase in the peak intensity upon increasing the concentration of the Ag NPs in the final matrix. The presence of the Ag Nps is obvious from the characteristic peaks of the Ag NPs at 38.1°, 44.2°, 64.7°, and 77.4°, corresponding to 111, 200, 220, and 311 facets, respectively. These data indicate the formation of the final composite as suggested.

Fig. 1b represents the FT-IR spectra of Fe_3_O_4_/HAP/PrNH_2_ and Fe_3_O_4_/HAP/PrNHCS_2_H. The FT-IR spectrum of the magnetic HAP shows the characteristic bands of both HAp and Fe_3_O_4_. The peak at 560 cm^−1^ shows the formation of the Fe–O band and the presence of the surface hydroxy groups is revealed by the peaks at 1642 and 3425 cm^−1^. Also, these spectra contain the characteristics of HAp as well. The peaks at 570 and 1040 cm^−1^ correspond to the presence of PO_4_^3−^. The peak around 1500 cm^−1^ corresponds to the presence of the CO_3_^2−^ in the composite. The wide peaks and small sharp peaks around 3500 cm^−1^ indicate the presence of the OH groups in the structure of HAP. In the case of Fe_3_O_4_/HAP/PrNH_2_, the wide peak at 3500 intensified, clearly indicating the formation of the amine groups over the surface of the composite. The peak at 1000–1150 shows a small change of character, which can be due to the addition of the Si–O–Si bonds to the composite. New peaks around 2900 cm^−1^ emerged which proves the presence of the aliphatic chain in the composite. In the case of Fe_3_O_4_/HAP/PrNHCS_2_H, the intensity of the wide peak at 3500 cm^−1^ dramatically decreased which proves the formation of the dithiocarbamate over the amine groups.

A vibrating-sample magnetometer (VSM) analysis was conducted to study the magnetic properties of the proposed composite. Fig. 1 c shows the magnetization saturation curves of the bare and the modified Fe_3_O_4_. The bare Fe_3_O_4_ NPs show high super paramagnetic properties with a saturation magnetization value (SMV) of 32.8 emu g^−1^, indicating the high synthesis quality of the NPs. However, the magnetization saturation curve of the Fe_3_O_4_/HAP/PrNHCS_2_H.Ag_NPs_ shows a drastic decrease in the SMV to 4.7 emu g^−1^. This drop in the SMV is due to the shielding effect of the dithiocarbamate-decorated HAP shell, further proving the successful formation of the composite.

Finally, to ensure the elemental composition of the final composite, the EDS analysis was conducted, which shows the presence of Fe, O, Ca, P, Si, N, S, and Ag (Fig. 1d). These data also prove the trend of the loading percentage of the Ag NPs as suggested. However, the actual loading of the Ag NPs was slightly lower than the theoretical suggestions. This trend shows that the unattached Ag NPs were separated from the sample during the washing step, which helps to avoid leaching. For simplification, the theoretical amounts are used in this manuscript to describe the samples.

**Fig. 2:**
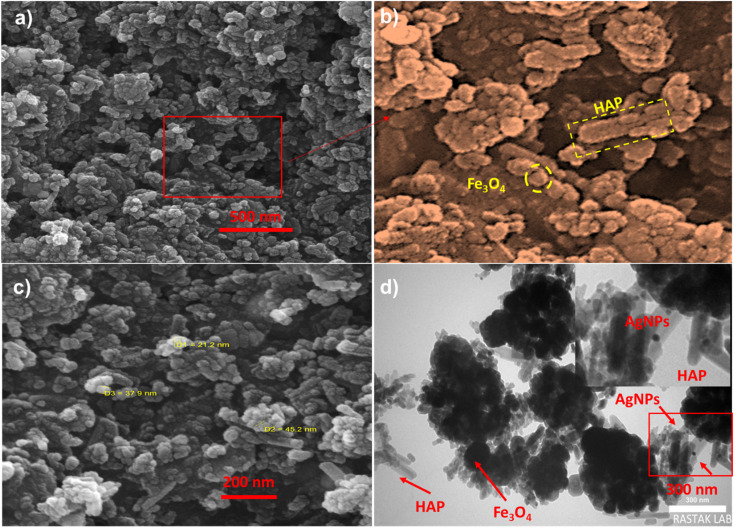
Fe_3_O_4_/HAP/PrNHCS_2_H.Ag_NPs_: (a and c) The SEM image of the Fe_3_O_4_@HAp-NH/CS_2_@Ag_NPs_ and its magnified image (b). (d) TEM image of the Fe_3_O_4_/HAP/PrNHCS_2_H.Ag_NPs_.

Fig. 2a–c show the SEM images of the Ag NP decorated dithiocarbamate-modified magnetic HAP. As can be seen, the modified Fe_3_O_4_/HAP mostly adapts the spherical morphology of the bare Fe_3_O_4_ core, with a mean diameter of 300 nm. Moreover, some particles with whisker-like morphology can be observed alongside the magnetite NPs, indicating the presence of the HAP NPs in the final composite. These data are further confirmed by TEM images (Fig. 2d), where the presence of the magnetic core with a mean diameter of 300 nm covered with nano-sized whisker-like HAP particles is evident. This is important since previous studies show that nano-sized HAP is more desirable for bio-related applications.^24,25^ Additionally, the TEM images also show the presence of the mono-disperse Ag NPs over the surface of the magnetic composite.

## Antibacterial activity of Fe_3_O_4_/HAP/PrNHCS_2_H.Ag_NPs_

The results of antibacterial effects (MIC and MBC values) of the Fe_3_O_4_/HAP/PrNHCS_2_H.Ag_NPs_ nanocomposite and vancomycin against MRSA are given in Table 1. Our study showed that the Fe_3_O_4_/HAP/PrNHCS_2_H.Ag_NPs_ nanocomposite has good antibacterial effects against methicillin-resistant *Staphylococcus aureus* isolates. So, the lowest value of MIC and MBC of the nanocomposite against MRSA1 was 0.29 ± 0.34 and 0.46 ± 0.33 μg mL^−1^ respectively. The obtained results were similar to the results of the study of Elbasuney *et al.*^26^ However, it showed a better antibacterial effect than previous studies.^27–29^ This may be due to the uniform distribution of silver nanoparticles on the HAP surface. Therefore, due to its porous structure, HAP as a silver nanoparticle carrier can have durable antimicrobial properties with sustained release. The ionic form of Ag/HAP damages the bacterial cell wall that binds to nucleic acid (DNA and RNA) and prevents the replication of bacteria.^30^ The antibiotic vancomycin is considered a last choice for the treatment of severe infections caused by Gram-positive bacteria, including MRSA. Today, there are concerns about MRSA resistance to vancomycin.^31^ Therefore, if clinical trials are conducted, this compound can be proposed as an alternative antibiotic in the future.

**Table 1:** MIC and MBC values of Fe_3_O_4_/HAP/PrNHCS_2_H.Ag_NPs_ and the vancomycin antibiotic against MRSA isolates^a^

No. Bacterial isolates	Fe_3_O_4_/HAP/PrNHCS_2_H.Ag_NPs_		Vancomycin	
	MIC values (μg mL^−1^)	MBC values (μg mL^−1^)	MIC values (μg mL^−1^)	MBC values (μg mL^−1^)
MRSA1	0.29 ± 0.34	0.46 ± 0.33	0.5 ± 0.3	1.33 ± 0.43
MRSA2	0.33 ± 0.75	1 ± 0.61	0.81 ± 0.69	1.16 ± 0.65
MRSA3	0.37 ± 0.75	1.37 ± 0.96	1.25 ± 1.03	1.66 ± 0.72
MRSA4	0.58 ± 1.55	2 ± 1.22	1.62 ± 1.38	1.66 ± 1.12
MRSA5	0.66 ± 1.94	2.25 ± 1.63	2.25 ± 1.63	3.33 ± 1.10
MRSA6	1.16 ± 2.22	3.5 ± 1.65	2.75 ± 1.92	3.33 ± 1.44
MRSA7	1.66 ± 2.41	3.5 ± 2.3	3.5 ± 2.29	5.33 ± 1.79
MRSA8	3.33 ± 2.24	6.5 ± 2.6	4.5 ± 2.17	13.3 ± 1.79
MRSA9	3 ± 2.94	7.25 ± 1.92	6.25 ± 2.27	18.6 ± 9.66
MRSA10	6.66 ± 2.21	18.5 ± 8.17	7.5 ± 2.17	26.6 ± 9.91
Control MRSA	1.3 ± 0.47	2.6 ± 0.94	3.3 ± 0.94	6.6 ± 1.8

a Values are means ± SD (*n* = 3).

The antibiofilm effects of this compound against *Staphylococcus aureus* (ATCC 35556) and *Pseudomonas aeruginosa* (ATCC 15442) showed that the Fe_3_O_4_/HAP/PrNHCS_2_H.Ag_NPs_ nanocomposite has high efficiency in inhibiting biofilm formation in Gram-positive and Gram-negative bacteria. This effect was significant against the Gram-positive bacteria-producing biofilm as mentioned in Table 2. Previous studies also confirm these results.^26,32,33^ In previous studies, the concentrations of HAP NPs to inhibit biofilm formation were different. So, in the Elbasuney *et al.* study,^26^ the concentration of 50 μg mL^−1^, and in the James *et al.* study (i), the concentration of 10 mg mL^−1^ inhibited biofilm production. In the current study, concentrations of 0.5 and 2 were able to inhibit biofilm production in *Staphylococcus aureus* and *Pseudomonas aeruginosa* bacteria, respectively. The difference in the percentage of inhibition in different studies is due to several components such as the high potential of antimicrobial agents to bind to the surface due to the increase in the surface area of the Ag-HAP nanocomposite and the particle size, as well as the penetration mode and the effect of the properties of different chemicals and the interaction of the Ag-HAP nanocomposite with biofilm-producing bacteria are related.^34^ Therefore, in addition to inhibiting biofilms on hospital surfaces, especially in intensive care rooms, this compound can also be used in the treatment of joint and bone infections and implant implantation.

**Table 2:** MICs values of Fe_3_O_4_/HAP/PrNHCS_2_H.Ag_NPs_ to inhibit biofilm formation^a^

Antibacterial material	*Pseudomonas aeruginosa* ATCC 15442	*Staphylococcus aureus* ATCC 35556
	MICs	MICs
Fe_3_O_4_/HAP/PrNHCS_2_H.Ag_NPs_	0.66 ± 0.23	0.2 ± 0.05
Ciprofloxacin	0.33 ± 11	0.5

a Values are means ± SD (*n* = 3).

## Toxicological study of Fe_3_O_4_/HAP/PrNHCS_2_H.Ag_NPs_

**Fig. 3:**
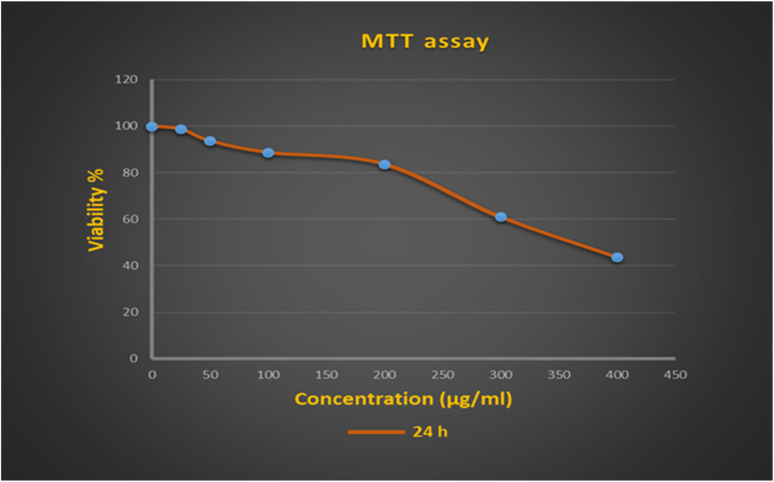
Viability rates of the NIH-3T3 cell line after 24 h of interaction with Fe_3_O_4_/HAP/PrNHCS_2_H.Ag_NPs_.

Cytotoxic effects of the synthesized nanocomposite on the NIH-3T3 cell line were evaluated using the MTT method. As seen in Fig. 3, the concentration of 25 has no toxic effect on the NIH-3T3 cell line. At concentrations of 50, 100, and 200 μg mL^−1^, cell viability was higher than 80% (Table 3), indicating that the Fe_3_O_4_/HAP/PrNHCS_2_H.Ag_NPs_ nanocomposite is not harmful to the cell line *in vitro*. Therefore, the nanocomposite was classified as mildly cytotoxic up to 200 μg mL^−1^ and qualified according to the ISO 10993–5:2016 standard. The concentrations used in the present work are higher than the concentrations used in previous studies. Moreover, the percentage of cell viability reported in the present study is relatively high.^35–37^ On the other hand, cytotoxicity was high at concentrations above 200 μg mL^−1^. iC50 was equal to 382 μg mL^−1^. This nanocomposite can be a suitable candidate for clinical trials due to its antibacterial effects at low concentrations and low cytotoxicity at high concentrations.

**Table 3:** Viability cell percentage after 24 h of contact with the Fe_3_O_4_/HAP/PrNHCS_2_H.Ag_NPs_

Cont	0 μg mL^−1^	25 μg mL^−1^	50 μg mL^−1^	100 μg mL^−1^	200 μg mL^−1^	300 μg mL^−1^	400 μg mL^−1^	IC50
NIH-3T3	100	98.6 ± 1.5	93.6 ± 1.15	88.6 ± 2.08	83.6 ± 3.78	61 ± 2.64	43.6 ± 2.1	382.37 ± 2.32

The anti-biofilm activity of the composites of silver nanoparticles has been investigated and confirmed in other studies, although silver nanoparticles themselves also have biofilm inhibition activity.^38–40^ Infection with bacterial biofilms due to their resistance to antibiotics is a global concern that poses health and economic challenges. On the other hand, up to 80% of bacterial infections in humans are related to biofilm-forming microbes.^41^ To treat biofilm-related infections, high concentrations of antibiotics, combined and sequential antimicrobial therapies, or adjuvants have been used in various studies.^42^ In addition, scientists are looking for alternative drugs, including nanoparticles, to solve this problem. Nanoparticles can deliver drugs in optimal concentrations and can enhance the efficacy of their treatment with fewer adverse effects.^43^ For this reason, the anti-biofilm activity of our synthesized materials was also investigated, which had promising results.

## Conclusion

In conclusion, here we prepared an Fe_3_O_4_/HAP/PrNHCS_2_H.Ag_NPs_ composite and used it as an antibacterial agent in an *in vitro* environment. Due to the strong affinity of the Ag NPs to the sulfur-containing functional groups, in this study, we modify the surface of the magnetic composite with dithiocarbamate groups to provide a suitable surface for the stabilization of the Ag NPs. The characterization studies show the mono-dispersity of the Ag NPs in the final composite and the formation and firmness of the magnetic core–shell Fe_3_O_4_/HAP/PrNHCS_2_H.Ag_NPs_. The as-prepared composite was employed as a bactericidal and antibiofilm agent against the MRSA, showing promising results. Moreover, the cell assay experiments showed low toxicity of the composite toward NIH-3T3 cells. Overall, this compound can be proposed as an alternative antibiotic in the future.

## Data availability

All data generated or analysed during this study are included in this published article.

## Conflicts of interest

There are no conflicts to declare.
